# Effects of intraoperative esketamine addition on gastrointestinal function after benign gynaecological laparoscopic surgery: a double-blind, randomized controlled study

**DOI:** 10.1186/s12871-023-02184-z

**Published:** 2023-06-23

**Authors:** Yuhua Ma, Ran Zhang, Xue Cao, Lin Zhang, Suozhu Bao, Jie Ren, Weiwei Ma

**Affiliations:** 1 Department of Anesthesiology, Xing’an Meng People’s Hospital, Inner Mongolia Autonomous Region, Xing’an Meng, Inner Mongolia 137400 China; 2 Department of Anesthesiology, Peking University People’s Hospital, Beijing, 100044 China

**Keywords:** Esketamine, Opioid-sparing anaesthesia, Laparoscopic surgery, Benign gynaecological lesions, Gastrointestinal function

## Abstract

**Background:**

Gastrointestinal hypokinesis can occur transiently after benign gynecologic surgery. Opioids cause the side effect of postoperative gastrointestinal hypokinesis, but an opioid-sparing anaesthetic protocol based on esketamine reduces intraoperative opioid consumption. Therefore, this study hypothesised that an opioid-sparing anaesthetic protocol based on esketamine would shorten the gastrointestinal function recovery time after benign gynaecological laparoscopic surgery.

**Methods:**

This was a prospective randomized controlled double-blind study conducted in a single centre. All patients scheduled for elective benign laparoscopic gynaecological surgery at Xing’an Meng People’s Hospital, Inner Mongolia Autonomous Region, from November 2021 to April 2022 were consecutively enrolled and randomly divided into the opioid-sparing anaesthesia group (Group OS) and the conventional anaesthesia group (Group C). Postoperative first exhaust time, feeding time and postoperative nausea and/or vomiting (PONV) were analyzed in both groups.

**Results:**

A total of 71 patients were enrolled in this study, including 35 in Group OS and 36 in Group C. The general condition, operative time, type of surgery, intraoperative bleeding, intraoperative fluid volume and intraoperative urine volume were not statistically different between the two groups. Compared with Group C, significantly shorter first postoperative flatus time (11 [8, 14] h vs. 14 [11, 18], *p* = 0.003) and anaesthesia resuscitation time (7 [6, 9] h vs. 9 [7, 11] h, *p* = 0.013)were observed in the OS group. The incidence of PONV in Group OS was significantly lower compared with Group C (11.4% vs. 41.7%, *p* = 0.007).

**Conclusion:**

The esketamine-based opioid-sparing anaesthetic protocol can shorten the postoperative first flatus time after benign laparoscopic surgery in gynaecology, and reduce the incidence of PONV. In addition, the application of esketamine may reduce the postoperative opioid dose requirement of patients.

**Trial registration:**

: This study was registered with the China Clinical Trials Registry (registration number: ChiCTR2100052528, 30/10/2021).

## Introduction

Benign gynecologic surgery, including laparoscopic surgery, is associated with transient gastrointestinal hypokinesis leading to postoperative intestinal obstruction (POI) [1, 2]. For gynecologic surgery, the incidence of this benign surgical POI is approximately 7–18% [1]. If POI persists for a longer period, additional postoperative complications may develop such as delayed surgical wound healing, pulmonary atelectasis, pneumonia and deep vein thrombosis, prolonged hospitalisation and increased medical costs [3–7].

Previous studies have shown that the incidence of POI is associated with operative time, intraoperative blood loss, surgical trauma, intestinal manipulation, and opioid consumption [5–8]. Opioid consumption is a modifiable risk factor for POI, which can be decreased by anesthesiologists. Therefore, opioid-sparing anaesthesia protocols have become an increasing trend in anaesthesia.

Opioid-sparing anaesthesia is implemented by partially replacing the effects of opioids with non-opioid analgesics or regional blocks [9]. As a non-competitive N-methyl-D-aspartate receptor (NMDA) antagonist, esketamine shows a good analgesic effect, supporting its use as an analgesic alternative to opioids in opioid-sparing anaesthesia [10]. Whereas the potential of esketamine to improve postoperative depression and reduce hyperalgesia has been the focus of many previous studies, the effects of esketamine-based opioid-sparing anaesthesia protocol on postoperative gastrointestinal function have been less studied. This study hypothesised that an esketamine-based opioid-sparing anaesthetic protocol could shorten the recovery time of gastrointestinal function after benign laparoscopic surgery in gynaecology.

## Methods

This prospective randomized double-blinded clinical trial, which adhered to CONSORT guidelines, was approved and was performed from November 2021 to April 2022, in accordance with the Helsinki Declaration of the World Medical Association. The study was reviewed and approved by the Medical Ethics Committee of Xi’an League People’s Hospital, Inner Mongolia Autonomous Region (approval number: YJXM2021YB2, 25/09/2021, Chairman: Hui Jiang) and registered with the China Clinical Trials Registry (registration number: ChiCTR2100052528, 30/10/2021). All patients signed an informed consent form.

All patients aged 18–65 years scheduled to undergo elective benign laparoscopic gynecologic surgery at Xing’an Meng People’s Hospital, Inner Mongolia Autonomous Regio were consecutively enrolled. The inclusion criteria were as follows: (1) American Society of Anesthesiologists (ASA) class I or II patient; (2) undergoing benign laparoscopic gynaecological surgery, and (3) consenting voluntarily to participate in the study and signing an informed consent form. The exclusion criteria were as follows: (1) pregnant women; (2) patients categorised in ASA class III and above; (3) emergency surgery; (4) legally protected adults (under judicial protection, guardianship or supervision) deprived of liberty; (5) history of laparotomy, and (6) allergy to experimental drugs.

### Blinding and randomisation method

In this study, patients were randomly grouped using computer-generated random numbers, and the results of the grouping were kept in sealed opaque envelopes. After obtaining informed consent from the patients, the nurse opened the envelope and divided the patients into either the opioid-sparing anesthesia group (OS group) or the conventional anesthesia group (C group). At the same time the nurse prepared induction medication and intraoperative maintenance medication. Preparation of anesthesia induction medication: 100 mg esketamine and 50 µg sufentanil were configured as 10 ml solution placed in a 10 ml syringe, respectively, and labeled as experimental medication provided to the anesthesiologist in the operating room and given at 0.05 ml.kg-1 during induction. Maintenance drug configuration: In the OS group, 50 mg of esketamine was configured as 50 ml and placed in a 50 ml syringe, and in the C group, 50 ml of 0.9% sodium chloride was placed in a 50 ml syringe, and both were labeled as experimental drugs and provided to the anesthesiologist in the operating room, and intraoperative maintenance was continuously pumped at 0.25 ml.kg-1.h-1. Postoperative follow-up was done by a nurse who was unaware of the grouping. The interoperative anesthesiologists, patients, and postoperative medical staff in this study were unaware of the grouping.

### Anaesthesia method

All patients fasted for 6–8 h and abstained from drinking for 4 h. After patients entered the operating room, intravenous access was routinely opened, oxygen was administered by face mask, and heart rate, noninvasive blood pressure, bispectral index (BIS), respiration, and oxygen saturation were continuously monitored.

Group OS: Propofol 1.5–2.5 mg/kg, esketamine 0.5 mg/kg and cis-atracurium 0.15–0.20 mg/kg were used for induction, and a laryngeal mask was placed after successful induction. Intraoperatively, esketamine 0.25 mg.kg^− 1^.h^− 1^ was continuously pumped, and the pumping was stopped at the beginning of the wound closure.

Group C: Propofol 1.5–2.5 mg/kg, sufentanil 0.25 µg/kg and cis-atracurium 0.15–0.20 mg/kg were used for induction, and a laryngeal mask was placed after successful induction.

Propofol and remifentanil were used intraoperatively in both groups. The propofol dosage was adjusted under BIS monitoring to target a BIS value of between 45 and 60. The starting dose of remifentanil was 6 µg.kg^− 1^.h^− 1^. The dose was adjusted at rate of 1 µg/kg/h under monitoring of change in heart rate and blood pressure. When the remifentanil dose was more than 9 µg.kg^− 1^.h^− 1^, 0.1 µg/kg sufentanil was given intermittently. The change in heart rate and blood pressure was maintained between 80% and 120% of the basal level. Intraoperatively, cis-atracurium 0.03 mg/kg was given intermittently to maintain the muscle relaxation status.

Propofol and remifentanil were stopped immediately after surgery and sufentanil 0.1 µg/kg and ketorolac 30 mg were given intravenously. All patients were given peri-incisional infiltration by the surgeon before skin suturing and 15–20 ml of 0.5% ropivacaine (5 ml/incision) was used as a local anaesthetic. All patients were given a 5-HT3 receptor antagonist for prophylactic antiemetic before the end of the surgery. All patients were admitted to the post-anaesthesia recovery room (PACU) with a laryngeal mask after surgery for recovery from anaesthesia. After successively achieving spontaneous ventilation and consciousness, the laryngeal mask was removed and the patients were transferred to the general ward. All patients were allowed to drink water starting 6 h after surgery. All patients were routinely given oral ibuprofen extended-release capsules 0.3 g/12 h postoperatively for postoperative analgesia. If the numerical rating scale of pain (NRS) > 3 points, tramadol or fentanyl or morphine was given intravenously for analgesic rescue.

### Outcomes and data collection

The primary endpoint of this study was the time of first postoperative flatus. Secondary endpoints were anaesthesia resuscitation time, postoperative first feeding time, postoperative nausea and/or vomiting (PONV) incidence, dizziness, postoperative numerical rating scale (NRS) of pain and hospital days postoperatively. All these data were collected by anaesthesia nurses blinded to the subgroups.

Resuscitation time was defined as the time between cessation of anaesthetic drug infusion and removal of the laryngeal mask. Postoperative feeding time was defined as the time between the patient’s return to the ward and the time of semi-liquid postoperative feeding. PONV was defined as the occurrence of nausea and/or vomiting from the end of surgery to postoperative day 2. The follow-up nurse assessed the patient’s pain on postoperative days 1, 2, and 3 at 4 pm and recorded the patient’s pain NRS score (0 = no pain, 10 = the most severe pain) at the follow-up visit.

In addition to the primary and secondary endpoints of the study, the patients’ general information (age, height, weight, ASA classification, smoking history and history of previous abdominal surgery), intraoperative conditions (operative time, anaesthesia time, intraoperative sufentanil consumption, intraoperative remifentanil consumption, intraoperative fluid volume, intraoperative blood transfusion and intraoperative urine volume), type of surgery and postoperative recovery (mental status and analgesic rescue) were collected. Mental status was only subjectively assessed based on family members’ reports on whether the patient’s mental status changed before and after surgery. The analgesic rescue was recorded irrespective of whether it was applied or not but the type and dose of medication were not recorded.

### Statistical analysis

Based on the results of the pretest, the time to first postoperative expulsion was 17.2 ± 6.9 h for patients receiving the opioid-sparing anaesthesia protocol and 27.1 ± 16.2 h for patients receiving the conventional anaesthesia protocol. Taking a test efficacy of 90% and α as 0.05, a sample size of 35 cases per group was obtained using PASS15 software (NCSS LLC, Utah, USA). Considering a 10% nonresponse, 39 cases per group were required.

Categorical data were expressed as percentages, and the χ2 test was used for comparison between groups. Continuous data that conformed to a normal distribution were expressed as mean ± SD, and independent samples t-test was used for comparison between groups. Continuous data that did not conform to a normal distribution were expressed as median [25% quartiles, 75% quartiles], and group comparisons were performed using the Mann-Whitney U test or the Kruskal-Wallis test. All statistical analyses were performed using SPSS 25.0 (IBM Corp., Armonk, NY, USA). *P* < 0.05 (two-sided) was considered statistically significant.

## Results

**Fig. 1:**
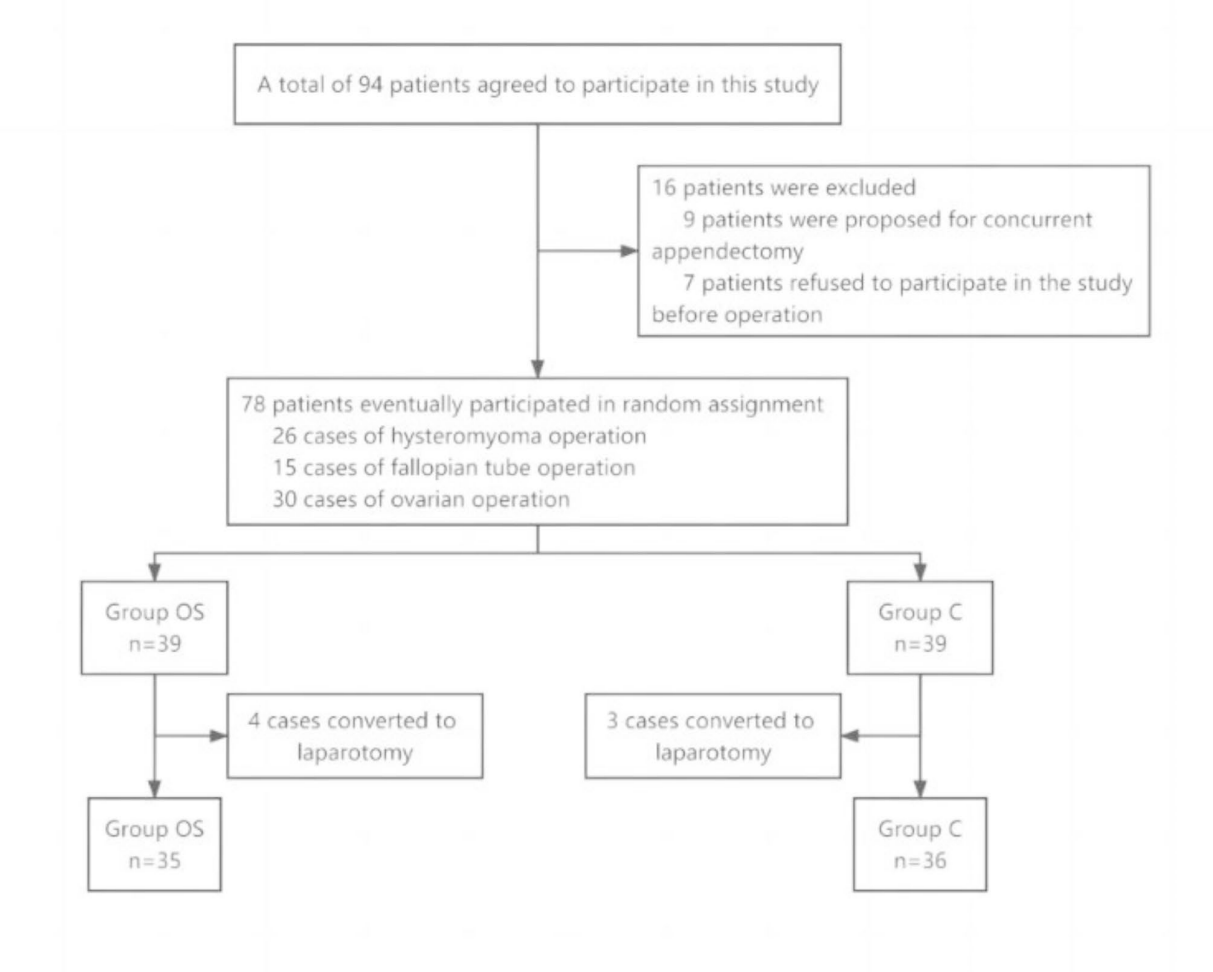
The study flow chart Notes: OS group, opioid-sparing anaesthesia; C group, conventional anaesthesia group

A total of 94 consenting patients participated in this study from November 2021 to April 2022 at Xing’an Meng People’s Hospital, Inner Mongolia Autonomous Region. Among them, 9 patients had their surgical plan changed to gynaecological surgery with appendectomy on the day of surgery and 7 preoperative patients declined to participate in the study. Therefore, a total of 78 patients were enrolled in the study and were randomly divided into 2 groups of 39 patients each. Among them, 4 patients in Group OS and 3 patients in Group C were changed to laparotomy due to severe abdominal adhesions. Therefore, data for 35 patients in Group OS and 36 patients in Group C were finally analyzed (Fig. 1).

**Fig. 2:**
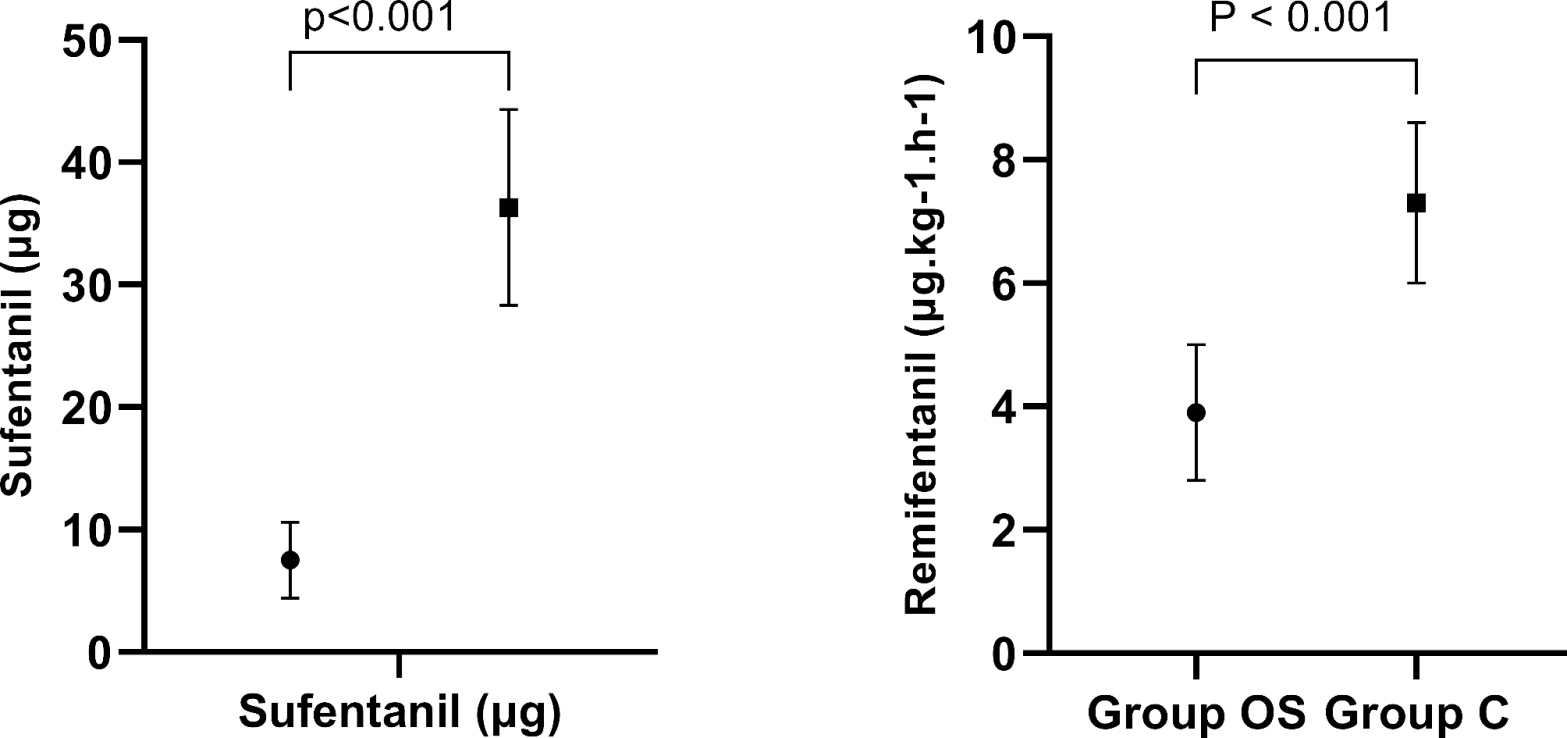
Comparison of intraoperative sufentanil and remifentanil dosage Notes: OS group, opioid-sparing anaesthesia; C group, conventional anaesthesia group

There were no significant differences between the two groups in age, height, weight, ASA score, smoking history, history of previous abdominal surgery, intraoperative fluid volume, urine volume, bleeding volume, type of surgery, duration of anaesthesia and duration of the operation (*p* > 0.05) (Tables 1 and 2). Compared with Group C, intraoperative sufentanil consumption and intraoperative remifentanil consumption were significantly lower in Group OS (7.5 ± 3.1 µg vs. 36.3 ± 8.0 µg, *p* < 0.001 and 3.9 ± 1.1 µg.kg^− 1^.h^− 1^ vs. 7.3 ± 1.3 µg.kg^− 1^.h^− 1^, *p* < 0.001, respectively (Fig. 2).

**Table 1:** Comparison of general conditions between OS and C groups

	OS group (n = 35)	C group (n = 36)
Age (year), mean ± SD	40 ± 7	39 ± 8
Height (cm), mean ± SD	161 ± 5	158 ± 15
Body weight (kg), mean ± SD	64 ± 11	65 ± 19
ASA (1/2)	19/16	21/15
Smoking history, n (%)	6 (17.1)	4 (11.1)
History of previous abdominal surgery, n (%)	16 (45.7)	15 (41.7)

Notes: OS group, opioid-sparing anaesthesia; C group, conventional anaesthesia group

**Table 2:** Comparison of intraoperative conditions between OS and C groups

	OS group (n = 35)	C group (n = 36)	P
Intraoperative infusion volume (ml), median[IQR]	600 [600,800]	650 [600,800]	0.890
Intraoperative urine volume (ml), median[IQR]	100 [50,200]	100 [50,138]	0.595
Intraoperative blood loss (ml), median [IQR]	20 [10, 30]	20 [10, 30]	0.588
Anesthesia time (min), mean ± SD	108 ± 39	92 ± 34	0.067
Operation time (min), mean ± SD	79 ± 39	64 ± 33	0.096
Type of surgery			0.618
Uterine fibroid surgery	13	13	
Fallopian tube surgery	9	6	
Ovarian surgery	13	17	

Notes: OS group, opioid-sparing anaesthesia; C group, conventional anaesthesia group

Compared with Group C, the resuscitation time and first postoperative flatus time were significantly shorter in Group OS (7 [6, 9] h vs. 9 [7, 11] h, *p* = 0.013) and 11 [8, 14] h vs. 14 [11, 18] h, *p* = 0.003, respectively). There was no statistical difference between the 2 groups in postoperative feeding time and the duration of postoperative hospitalization (*p* > 0.05) (Table 3).

**Table 3:** Comparison of main results between OS and C groups

	OS group (n = 35)	C group (n = 36)	P
Resuscitation time (min), median [IQR]	7 [6, 9]	9 [7, 11]	0.013
First postoperative flatus time (h), median [IQR]	11 [8, 14]	14 [11, 18]	0.003
Postoperative feeding time (h), median [IQR]	18 [15, 21]	19 [17, 21]	0.201
Postoperative hospital stay (d), median [IQR]	4 [4, 5]	4 [4, 5]	0.858

Notes: OS group, opioid-sparing anaesthesia; C group, conventional anaesthesia group

There was no significant difference in postoperative NRS scores and 24-hour postoperative analgesic rescue between the 2 groups (*p* > 0.05). Compared with Group C, the incidence of PONV was significantly lower in Group OS (11.4% vs. 41.7%, P = 0.007). The incidence of postoperative dizziness was not significantly different between the 2 groups (17.1% in Group OS vs. 38.9% in Group C; Table 4). No postoperative psychiatric abnormalities occurred in the 2 groups.

**Table 4:** Comparison of postoperative analgesia and adverse reactions between OS and C groups

	OS group (n = 35)	C group (n = 36)	P
NRS, median[IQR]			
POD1	1 [1, 2]	1[0,2]	0.278
POD2	1 [1, 2]	1 [1, 2]	0.718
Analgesic rescue, n (%)	4 (11.4)	7 (19.4)	0.514
Tramadol	3 (8.6)	5 (13.9)	
Fentanyl	1 (2.9)	2 (5.6)	
Morphine	0 (0)	0 (0)	
PONV, n (%)	4 (11.4)	15 (41.7)	0.007
Dizziness, n (%)	6 (17.1)	14 (38.9)	0.064

Notes: NRS, numerical rating scale of pain; POD, postoperative day; PONV, postoperative nausea and/or vomiting; OS group, opioid-sparing anaesthesia; C group, conventional anaesthesia group

## Discussion

In this study, we found that an esketamine-based opioid-sparing anaesthetic protocol could shorten the recovery time of gastrointestinal function after benign gynaecological laparoscopic surgery and reduce the incidence of PONV, but had no significant effect on the postoperative feeding time and duration of postoperative hospitalisation.

The recovery of postoperative gastrointestinal function, which is commonly assessed using time to flatus, time to defecation, bowel sounds, and imaging, is an important prognostic factor for POI patients [2, 3, 8, 11, 12]. However, as the time of first postoperative flatus is the most commonly used method [3], this index was also used in this study. The recovery of postoperative gastrointestinal function is influenced by various factors, such as the surgical approach, operative time, surgical trauma, intraoperative blood loss, intestinal manipulation, intraoperative fluid volume, postoperative feeding time and postoperative activity time, and perioperative opioid consumption [8, 12, 13]. Among them, opioid consumption is the most concerning factor for anesthesiologists [13, 14].

Opioid consumption is strongly associated with impaired gastrointestinal motility and postoperative intestinal obstruction. Studies have found higher opioid consumption in patients with intestinal obstruction compared with patients without intestinal obstruction [13, 14]. Opioids exert a dose-dependent inhibitory effect on intestinal motility due to the presence of several opioid receptor types in the intestine, such as κ, µ, and δ. Among them, κ and µ receptor agonists modulate cholinergic transmission in the mesenteric plexus [15, 16]. All three subtypes of opioid receptors have been identified in the submucosal and intramuscular plexus neurons. In humans, mu-opioid receptors are present on immune cells in the lamina propria, submucosa, and interosseous neurons. In addition to the direct effects of opioids on gastrointestinal motility, opioids may also affect fluid transport and produce antisecretory effects. Following intravenous, intramuscular and epidural administration of opioids, inhibitory effects are detected in gastrointestinal function [15, 16]. The use of an opioid-sparing anaesthetic protocol reduces intraoperative opioid consumption, thereby mitigating the effects of opioids, which in turn facilitates postoperative recovery of gastrointestinal function.

Reducing opioid consumption is an increasing practice in anaesthesia. Epidural combined with general anaesthesia is the most commonly used opioid-sparing anaesthetic protocol in abdominal surgery [17–20]. Previous studies have shown that epidural anaesthesia can effectively reduce perioperative opioid consumption in gynecologic surgery and postoperative pain [17, 21]. However, because of side effects, such as hypotension, lower extremity muscle weakness and urinary retention, epidural anaesthesia is not widely used in gynecologic surgery [22, 23]. Moreover, some studies have shown that epidural anaesthesia does not have a decisive advantage over general anaesthesia in gynecologic surgery [24, 25]. The other protocols for opioid-sparing anaesthesia include transversus abdominis plane block (TAP) or lumbar square block (QLB) combined with general anaesthesia. TAP is widely used because it is simple to perform, but it does not reduce intraoperative opioid consumption and does not have an absolute analgesic advantage over postoperative wound infiltration in gynecologic patients [26]. QLB has the potential to be widely used as a local anaesthetic and may also have a visceral analgesic effect. A randomized controlled trial (RCT) found that post-QLB significantly improved postoperative gynecologic laparoscopic pain for up to 24 h and reduced intraoperative opioid use compared with a placebo [27]. However, QLB is complex and not available to all anesthesiologists. Therefore, opioid-sparing anaesthesia via non-opioid analgesics is a convenient option.

Tu et al. found that propofol combined with esketamine had good safety and reliability in the induction of anaesthesia, improved hemodynamics, improved surgical stress and inflammatory response, shortened anaesthesia time, and promoted postoperative cognitive recovery [10]. A meta-analysis by Wang et al. showed that low-dose esketamine combined with sufentanil for spinal fusion patients not only improved postoperative analgesia but also reduced the need for opioids and reduced the incidence of postoperative nausea and vomiting and delayed recovery of gastrointestinal function, without affecting the time to resuscitate [28]. In this study, the use of an esketamine-based opioid-sparing anaesthetic protocol resulted in faster resuscitation after general anaesthesia, shorter postoperative gastrointestinal recovery time, and reduced incidence of PONV, which are consistent with previous studies.

A postoperative analgesic protocol of wound infiltration plus oral NSAIDs was used for benign gynecologic laparoscopic surgery in our centre. Previous studies have shown that wound infiltration provides good postoperative analgesia in patients undergoing gynecologic laparoscopy [19]. Similarly, oral NSAIDs drugs have also been shown to provide good analgesia in postoperative analgesia in gynecologic laparoscopic patients [29, 30]. In this study, the follow-up of postoperative analgesia found satisfactory analgesia in both groups.

The analgesic rescue was required in 19.4% of patients in the conventional anaesthesia group at 24 h postoperatively compared with 11.4% in the opioid-sparing anaesthesia group. However, the difference between the two groups was not statistically significant. This may be explained that the application of esketamine reduced intraoperative sufentanil and remifentanil consumption. Yamashita et al. showed that large intraoperative applications of remifentanil can cause postoperative hyperalgesia [31]. Mauermann et al. confirmed that increased intraoperative fentanil consumption could increase postoperative opioid consumption and pain in patients. Although no studies have demonstrated that sufentanil causes postoperative hyperalgesia, since it belongs to the same fentanyl family, it is reasonable to conclude that sufentanil may also increase the risk of developing postoperative hyperalgesia. Reducing opioid consumption may reduce the incidence of hyperalgesia. Studies on postoperative hyperalgesia have also shown that the use of NMDA agonists such as ketamine could effectively reduce the incidence of postoperative hyperalgesia [32, 33], thereby improving acute postoperative pain and reducing opioid consumption. The present study used an esketamine-based opioid-sparing anaesthetic regimen that reduced intraoperative opioid consumption. Further esketamine application reduced the incidence of postoperative hyperalgesia, thus showing a difference in the 24-h postoperative analgesic rescue between the two groups. The lack of statistical difference between the two groups may be due to the small sample size, and further studies using larger samples are needed to verify this effect.

This study has several limitations. First, Although the type of rescue analgesic drugs was recorded in this study, the dose was not, and therefore may have influenced the initial results. Second, this study was a single-centre, small sample size study, and whether the protocol has the same effect on patients in other centres can only be confirmed by a larger multicenter study. Third, this study did not include a control group with different esketamine doses, and it remains to be studied whether smaller doses of esketamine can further reduce the incidence of adverse effects. Fourth, although the study observed the patients’ postoperative psychiatric condition, patients were not evaluated using an assessment scale. Therefore, further studies using a psychiatric assessment scale are needed to confirm whether the dosage of esketamine used in this study affects patients. Fifth, this study was limited to benign gynecologic laparoscopic surgery, and it is worthwhile to continue to investigate whether this protocol is feasible for other types of surgery and whether the effective dose of esketamine will change.

In conclusion, the esketamine-based opioid-sparing anaesthetic protocol can shorten the postoperative first flatus timerecovery time of gastrointestinal function after benign laparoscopic surgery in gynaecology, and reduce the incidence of PONV, and promote early recovery of patients. In addition, the application of esketamine may reduce the postoperative opioid dose requirement of patients.

## Data Availability

The datasets used and/or analysed during the current study are available from the corresponding author on reasonable request. Data supporting the results of this study are available from the Xing’an Meng People’s Hospital, Inner Mongolia Autonomous Region, but the availability of these data is restricted and they are used under licence from the current study and therefore not publicly available. However, data may be obtained from the authors upon reasonable request and with the permission of the Ethics Committee of the Xing’an Meng People’s Hospital.
